# An Allosteric Regulator of R7-RGS Proteins Influences Light-Evoked Activity and Glutamatergic Waves in the Inner Retina

**DOI:** 10.1371/journal.pone.0082276

**Published:** 2013-12-09

**Authors:** Matthew D. Cain, Bradly Q. Vo, Alexander V. Kolesnikov, Vladimir J. Kefalov, Susan M. Culican, Daniel Kerschensteiner, Kendall J. Blumer

**Affiliations:** 1 Department of Cell Biology and Physiology, Washington University School of Medicine, St. Louis, Missouri, United States of America; 2 Department of Ophthalmology and Visual Sciences, Washington University School of Medicine, St. Louis, Missouri, United States of America; Dalhousie University, Canada

## Abstract

In the outer retina, G protein-coupled receptor (GPCR) signaling mediates phototransduction and synaptic transmission between photoreceptors and ON bipolar cells. In contrast, the functions of modulatory GPCR signaling networks in the inner retina are less well understood. We addressed this question by determining the consequences of augmenting modulatory Gi/o signaling driven by endogenous transmitters. This was done by analyzing the effects of genetically ablating the R7 RGS-binding protein (R7BP), a membrane-targeting protein and positive allosteric modulator of R7-RGS (regulator of the G protein signaling 7) family that deactivates Gi/oα subunits. We found that R7BP is expressed highly in starburst amacrine cells and retinal ganglion cells (RGCs). As indicated by electroretinography and multielectrode array recordings of adult retina, ablation of R7BP preserved outer retina function, but altered the firing rate and latency of ON RGCs driven by rods and cones but not rods alone. In developing retina, R7BP ablation increased the burst duration of glutamatergic waves whereas cholinergic waves were unaffected. This effect on glutamatergic waves did not result in impaired segregation of RGC projections to eye-specific domains of the dorsal lateral geniculate nucleus. R7BP knockout mice exhibited normal spatial contrast sensitivity and visual acuity as assessed by optomotor reflexes. Taken together these findings indicate that R7BP-dependent regulation of R7-RGS proteins shapes specific aspects of light-evoked and spontaneous activity of RGCs in mature and developing retina.

## Introduction

Signal transduction by G protein-coupled receptors (GPCRs) in the outer retina converts visual stimulation ultimately to patterns of activity of retinal ganglion cells (RGCs). Visual opsins trigger light-evoked activation of transducin, and Go-coupled type 6 metabotropic glutamate receptors (mGluR6) regulate transmitter release by ON bipolar cells [1], [2]. Gi/o-coupled receptors potentially modulate synaptic transmission in mature [3]–[7] and developing [8]–[10] retina. However, the diversity of these modulatory GPCRs has impeded progress toward understanding their functions in the inner retina.

Functions of specific G protein signaling networks in the retina recently have been probed by studying the consequences of augmenting signaling evoked by light or endogenous neurotransmitters. This approach has utilized mice lacking one or more members of the R7-RGS (regulators of G protein signaling) family (RGS6, 7, 9, 11), which accelerate G protein deactivation by functioning as GTPase-activating proteins (GAPs) specific for Gi/oα subunits [11]. Each R7-RGS isoform forms an obligate heterodimer with Gβ5 [12], [13] to regulate Gi/o signaling [11], [14]–[17] and has a distinct retinal expression pattern [18]–[20]. RGS9 is expressed in photoreceptor disk membranes where it deactivates transducin [21], [22]. RGS7 and 11 are expressed in ON bipolar cells and deactivate Goα, thereby facilitating light-evoked depolarization [23]–[28]. RGS6 and 7 are expressed in the inner retina [13], [19], [20], suggesting that further studies of the R7-RGS family may reveal new functions for Gi/o signaling in retina.

Because the absence of RGS7 and RGS11 or the entire R7-RGS family disorganizes dendritic arborization of ON bipolar cells [23], [28], these mutants are ill-suited to assess whether light-evoked responses in the inner retina are affected by augmenting Gi/o signaling. As an alternative, we hypothesized that R7-RGS function in the retina would be impaired rather than lost completely by disrupting R7 RGS-binding protein (R7BP), a palmitoylated SNARE-like protein that functions as a positive allosteric regulator of R7-RGS/Gβ5 heterodimers [17], [29]–[35]. R7BP is highly expressed in the inner retina [36]. It augments R7-RGS GAP activity [31], [37], enables RGS7 regulation of Giα [37], and facilitates recruitment of R7-RGS/Gβ5 complexes to GIRK channels [17]. However, R7BP ablation does not perturb outer retina organization, rod-driven activity, membrane association of RGS7, or protein expression of RGS6, RGS7, or RGS11 in the retina [36]. Therefore, ablation of R7BP may impair rather than eliminate activity of the R7-RGS protein family and modestly augment Gi/o signaling evoked by endogenous GPCR agonists in the inner retina.

Here we identify cell types in the inner retina that express R7BP and analyze the consequences of R7BP ablation. Our results provide evidence indicating that regulation of R7-RGS proteins by R7BP shapes specific aspects of light-evoked and spontaneous wave activity in the inner retina.

## Materials and Methods

### Animals

All animal procedures used protocols approved by the Washington University Animal Studies Committee (Protocol #20110184). R7BP^−/−^ mice produced by targeted deletion of exon 2 have been described previously [17]. R7BP^+/−^ mice were crossed six generations into the C57BL/6 background (Charles River Laboratories) and then interbred to produce WT and R7BP^−/−^ littermates of either sex for analysis.

### Tissue preparation and immunostaining

Mice were euthanized by CO_2_ asphyxiation followed by cervical dislocation. Enucleated eyes were fixed with paraformaldehyde (4%) in PBS (pH 7.4). Retinas were isolated in PBS and embedded in 4% low-melting point agarose. For fixation of brain, euthanized mice were perfused intracardially with PBS followed by paraformaldehyde (4%) in PBS. Brains were removed and post-fixed overnight at 4°C. Vertical retina and coronal brain slices were cut (60 µm and 100 µm, respectively) with a vibratome. Slices were blocked with 10% normal horse serum (NHS) in PBS and incubated with primary antibodies overnight in 5% NHS and 0.05% Triton X-100 (Sigma-Aldrich). The following antibodies were used: affinity purified rabbit anti-R7BP [38], goat anti-ChAT (#AB143 Millipore; 1∶100), goat anti-Brn3a (C20 Santa Cruz; 1∶500), Alexa Fluor 568-conjugated anti-rabbit and Alexa Fluor 488-conjugated anti-goat (Invitrogen; 1∶1000) antibodies. Slices were mounted using VectaShield (Vector Labs) and imaged with Olympus FluoView FV500 (Bakewell NeuroImaging Laboratory, WUSM). Retinal immunofluorographs were median filtered (1 pixel surround) using NIH Fiji [39].

### Electroretinography

Flash ERG measurements were performed with a UTAS-E3000 visual Electrodiagnostic System running EM for Windows (LKC Technologies). Mice (∼3 months old) were dark-adapted overnight. Under dim red illumination, mice were anesthetized with a cocktail of 80 mg/kg ketamine and 15 mg/kg xylazine. The body temperature of the mice was maintained at 37^o^C with a heating pad controlled by a rectal temperature probe. After positioning mice in the Ganzfeld dome, the recording electrodes (2.0 mm diameter platinum loops) were positioned on the corneal surface of each eye in a drop of 0.5% atropine sulfate (Bausch & Lomb) and 1.25% hydroxypropol methylcellulose (GONAK; Akorn Inc.). Reference and ground electrodes were placed at the vertex of the skull and back, respectively. For dark-adapted analysis, we recorded the responses to white light flashes of increasing intensity (–4.6 to 1.9 log cd s/m^2^) in total darkness. Mice were then light-adapted to a constant white background illumination of 2.3 log cd s/m^2^ for 10 min. Light-adapted responses were obtained to a series of light flashes (–0.01 to 2.67 log cd s/m^2^) in the presence of constant background illumination. At each intensity, responses to multiple trials were averaged. The a- and b-wave amplitude and latency were measured and quantified for comparison.

### Multielectrode array recordings of RGC activity

Mice were dark-adapted for at least 1 h prior to CO_2_ asphyxiation. For light-evoked recordings, eyes were enucleated and retinas were isolated under IR illumination. For spontaneous activity recordings, this step was performed under dim red illumination. During dissection, isolated retinas were maintained in cooled murine artificial cerebral spinal fluid (mACSF) (125 mM NaCl, 2.5 mM KCl, 1 mM MgCl_2_, 1.25 mM NaH_2_PO_4_-H_2_O, 20 mM glucose, 26 mM NaHCO_3_, 2 mM CaCl_2_) oxygenated with 95% O_2_, 5% CO_2_. Isolated retinas were placed ganglion cell layer down on a multi-electrode array (MEA), consisting of 252 electrodes at 100 µm spacing (MultiChannel Systems). To prevent movement of the retina, a transparent cell culture membrane (Corning) was placed over the retina and secured under a platinum ring. Retinas were allowed to equilibrate for 45–60 min before recording. While recording, retinas were superfused with oxygenated mACSF (30°C) at a rate of 1–1.5 mL/min.

### Visual stimulation

Stimuli were generated using an organic light emitting display (OLED) mounted in place of the condenser of an inverted light microscope (10x objective). Stimulation protocols were programmed using Matlab (Mathworks). Full field illumination (4 sec) was attenuated to 5 or 200 Rh*/rod/sec by neutral density filters placed between the display and objective. For checkerboard Gaussian white noise stimulation, the field was divided into squares (66 µm sides) and the intensity of the squares was chosen at random from Gaussian distribution with a constant mean and standard deviation at 40 msec intervals.

### Analysis of RGC light responses

Because MEA electrodes can record spikes from multiple RGCs, we used principal component analysis of waveforms (Offline Sorter, Plexon) to assign spike trains to individual RGCs. Individual RGCs were selected as those exhibiting spike trains in which less than 0.2% of interspike intervals were less than 2 msec. This analysis was restricted to transient ON RGCs. Firing rate was calculated by quantifying the number of spikes during illumination (5 sec bin). Latency was calculated as the time to reach maximum firing rate. To study RGC space-time receptive fields, we generated and analyzed spike-trigger averages (STA), as described in detail previously [40]. For STA analysis, retinas were stimulated with a Gaussian white noise checkerbox sequence. STA stimuli were the averages of the stimulus sequences (500 msec) preceding each spike for a given RGC. Because the quality of STAs depends on the number of spikes used to generated them, only RGCs with a robust total number of spikes were included for analysis (average number of spikes: WT: 4700±650, R7BP^−/−^: 4700±630). The temporal structure of the receptive field response was calculated from the average of the stimulus squares that have a standard deviation (SD) three-fold greater than the SD of background squares. Receptive fields were estimated as the radius of a 1-SD ellipse from a two-dimension Gaussian fit of the spatial profile at the STA temporal maximum. The radius of the receptive field was calculated as: r = √r_maj_r_min_, where r_maj_ and r_min_ are the major and minor axes. Time to peak was calculated as the time between maximum STA contrast and the spike.

### Analysis of RGC spontaneous wave activity

For recording of spontaneous waves, retinas were maintained in complete darkness for a recording period of 45 min. Individual RGCs were sorted as described above. Firing rate of RGCs was calculated as number of spikes at 5 sec bins. Burst duration was estimated by the width at half-maximum of a RGC's spike train autocorrelogram. Interwave interval was calculated as the average time between peaks in the population firing rate. To identify peaks, the firing rates of all RGCs from a retina were averaged and smoothed using an exponential filter: *y(t)*  =  *α* x *y(t*–*1)* + *(1*–*α)* x *x(t)*, where *α* is the degree of smoothing (0.9), *x(t)* is the mean firing rate, *y(t)* is the smoothed version. The running average of the firing rate, calculated using a Loess filter (*f*  =  0.67) multiplied by 1.5, was used as a threshold. Peaks were defined as the maximum point between two successive crossings of this threshold. Correlation indices were calculated as describe previously [41]: *CI_XY_*  =  [*N_XY_*(–*Δt*, +*Δt*) x *T*] / [*N_X(O,T)_* x *N_Y(O,T)_* x *2Δt*] where *N_XY_* is the number of spikes cell Y fired within ± *Δt* (0.1 sec) from spikes in cell X. *T* is the duration of the recording. *N_X(O,T)_* and *N_Y(O,T)_* are the total number spikes from cells X and Y, respectively.

### Anterograde labeling and analysis of retinogeniculate projections

Alexa Fluor-647 or Alexa Fluor-555 dye-conjugated cholera toxin B subunit (CTB) (1–2 µL, 2 mg/mL, Invitrogen) was injected intravitreally into opposite eyes of anesthetized P19 mice with a Picospritzer III (Parker). After 2 days, mice were euthanized and perfused, and brain slices were prepared as described above. Coronal brain slices (80 µm) were mounted and imaged at 10x magnification. Retinogeniculate segregation was quantified as described previously [42]. Briefly, images were background subtracted using rolling ball subtraction (200 pixels) and the area surrounding the dLGN was masked. R-values for each pixel were calculated in Matlab Software (MathWorks) as R  =  Log_10_(*Fi*/*Fc*), where *Fi* and *Fc* are the fluorescence of ipsilateral and contralateral channels, respectively. As a measure of segregation, the variance of R-values for the center four slices of each dLGN were averaged. To determine the area of contralateral and ipsilateral projections, background subtracted and masked images were thresholded (10%) and binarized. The total area of dLGN was set as the area of the dLGN mask and quantified using NIH Fiji, and the number of thresholded pixels for contralateral and ipsilateral projections were quantified and expressed as percentage of the total dLGN area. For area of overlap, thresholded images were merged. Merged pixels were isolated using NIH Fiji (RG2B Colocalization) and quantified as described above.

### Spatial vision measured by optomotor reflexes

For testing spatial vision, we measured optomotor responses in WT and R7BP^−/−^ mice (∼3 months old) using the OptoMotry virtual optomotor system (Cerebral Mechanics) [43], which utilizes a reflex in which mice move their heads to track a moving vertical sine wave grating. Mice were placed on a pedestal surrounded by computer monitors that displayed the grating and monitored with a video camera under normal or IR illumination. Stimuli were presented for a period of 5 sec before returning to 50% gray illumination. The protocol implemented a two-alternative, forced choice method in which the observer was blind to the direction of rotation of the grating and was forced to identify the direction based on the mouse's observed head movement [44]. A staircase paradigm was used for assessing contrast and spatial frequency thresholds, defined as a correct observer response of 70%. For determining contrast sensitivity, testing was performed under optimal tuning conditions in which spatial and temporal frequencies were set at 0.128 cyc/deg and 0.75 Hz, respectively [45]. Contrast sensitivity was defined as the inverse of the contrast at threshold [43]. For testing visual acuity, contrast (100%) and speed (5.4 deg/s) were kept constant, while spatial frequency was gradually increased. Visual acuity was defined as the spatial frequency at threshold. During testing, the observer was blind to the genotype of the animal. For scotopic (–4.5 log cd/m^2­^) testing, mice were dark-adapted overnight and neutral density film filters were placed between mice and the computer monitors. For photopic (1.8 log cd/m^2^) testing, mice were light adapted, and the acuity and contrast sensitivity examinations were repeated without filters.

### Statistics

Statistical analysis of electroretinograms was performed using repeated measures ANOVA, followed by Bonferroni’s (Dunn) post-test. Significance was determined before Bonferroni. Analysis of MEA data implemented a linear/generalized linear mixed model framework. This framework allows analysis of data from individual neurons, while still accounting for the correlated nature of the activity of neurons recorded from the same retina and during correlated wave activity. Because each type of data obtained from MEA experiments exhibited different statistical distributions, we determined which type of statistical distribution for the random error in the linear/generalized linear mixed model best fit the data. RGC receptive field radius was analyzed using linear mixed model with normally distributed random error. Light-evoked and wave spike rate and wave burst duration were analyzed similarly after log transformation. Log transformation did not result in a normal distribution of data measuring latency, STA time-to-peak, or interwave interval. In these cases, a generalized linear mixed model was performed, the random error had a gamma distribution for latency measurements and a negative binomial distribution for STA-time-to-peak and interwave interval measurements. Model assumptions were examined by residual plots and other model diagnostics. Homogeneity within retinas from the same genotype was assessed by intra-class correlation coefficient analysis. Student's t-test was used to determine significance of the optomotor response and retinogeniculate segregation data. Statistical significance was defined as p<0.05.

## Results

### R7BP is expressed highly in starburst amacrine cells and retinal ganglion cells

**Figure 1:**
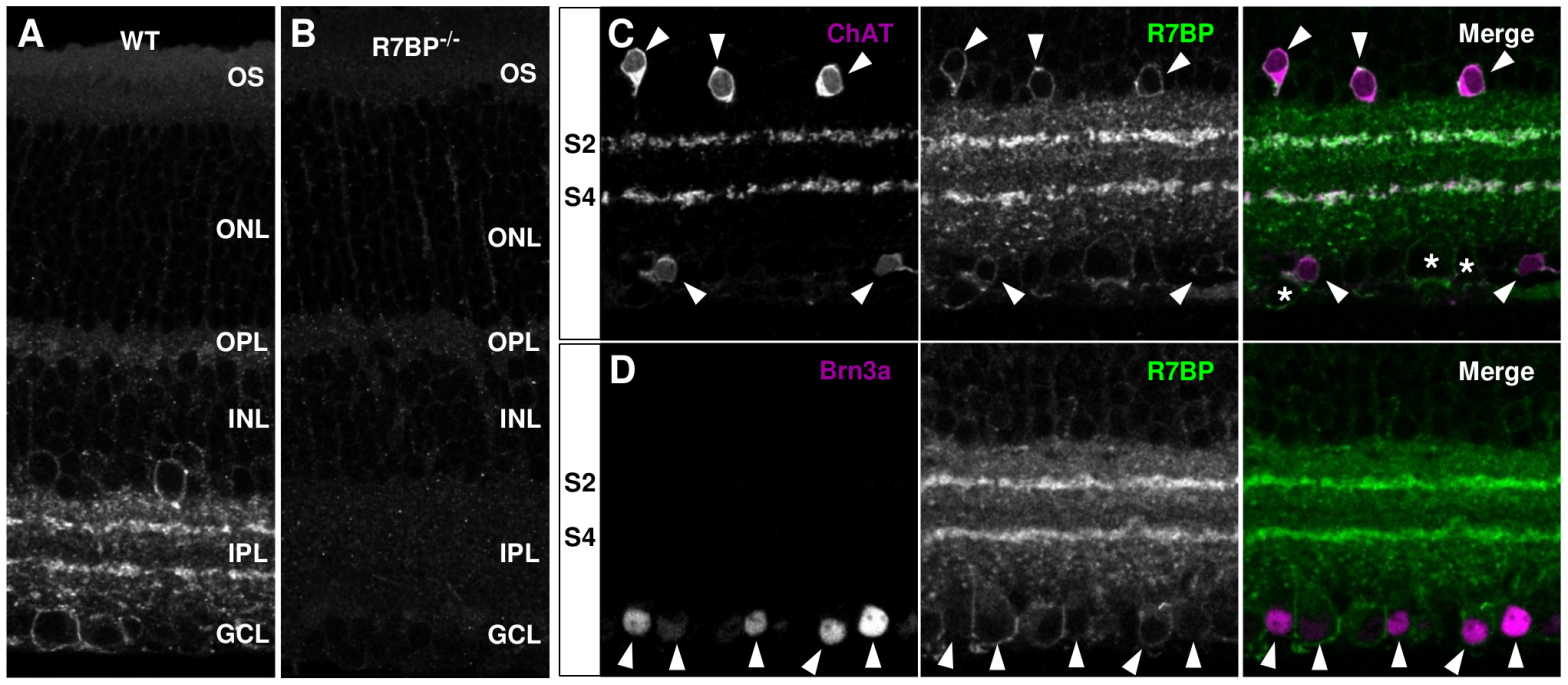
R7BP is expressed in starburst amacrine cells and Brn3a-positive RGCs. **A-B.** R7BP is expressed in the OPL, INL, IPL, and GCL in wild-type retina (A) as compared to R7BP^−/−^ retina (B). **C.** Expression of R7BP in starburst amacrine cells (SACs). Co-staining of the SAC marker (ChAT; magenta) and R7BP (green) in the S2/S4 sublaminae of the IPL and somata of the INL and GCL. Arrowheads mark ChAT-positive somata. Asterisks indicate R7BP-positive, ChAT-negative somata. **D.** Somatic expression of R7BP (green) in Brn3a-positive retinal ganglion cells (magenta). Arrowheads mark Brn3a-positive nuclei. Abbreviations are as follows: outer segment (OS), outer nuclear layer (ONL), outer plexiform layer (OPL), inner nuclear layer (INL), inner plexiform layer (IPL), ganglion cell layer (GCL).

To investigate roles of Gi/o signaling regulated by R7-RGS/Gβ5 complexes under the control of R7BP, we first identified retinal cell types that express R7BP by probing vertical retinal slices of adult mice with affinity-purified polyclonal R7BP antibodies [38]. Specific R7BP staining was expressed weakly in the outer plexiform layer (OPL) with stronger expression throughout the inner plexiform layer (IPL), especially in the S2 (OFF) and S4 (ON) sublaminae of the IPL, and in somata of the inner nuclear and ganglion cell layers (INL and GCL) (Figure 1A). No specific staining above background was observed in R7BP^−/−^ retinas, demonstrating antibody specificity (Figure 1B). Several results indicated that nearly all starburst amacrine cells (SACs) express R7BP. First, co-staining of R7BP and choline acetyltransferase (ChAT, a marker of SACs) was evident in S2 and S4, indicating the presence of R7BP in SAC processes. Second, R7BP also was expressed strongly on the somatic plasma membrane of most ChAT-positive cells in the INL (92%±2%, n = 3 retinas; Figure 1C, indicated by arrowheads) and a majority of displaced SACs (52%±1%) in the GCL. R7BP in the GCL also was detected on the somatic plasma membrane of some ChAT-negative cells (asterisks, Figure 1C). This suggests that R7BP is expressed in other cell types in the inner retina and is consistent with expression in other sublaminae of the IPL. Many of these ChAT-negative neurons were retinal ganglion cells (RGCs; 49% ± 3%; n = 3 retinas) as indicated by co-staining with Brn3a (an RGC marker; arrowheads in Figure 1D) [46]. Thus, the overall pattern of R7BP expression in retina is similar to the aggregate expression of RGS6, 7, and 11 [19], [20], [36], supporting the notion that R7BP regulates the function of one or more of these R7-RGS proteins in the inner retina.

**Figure 2:**
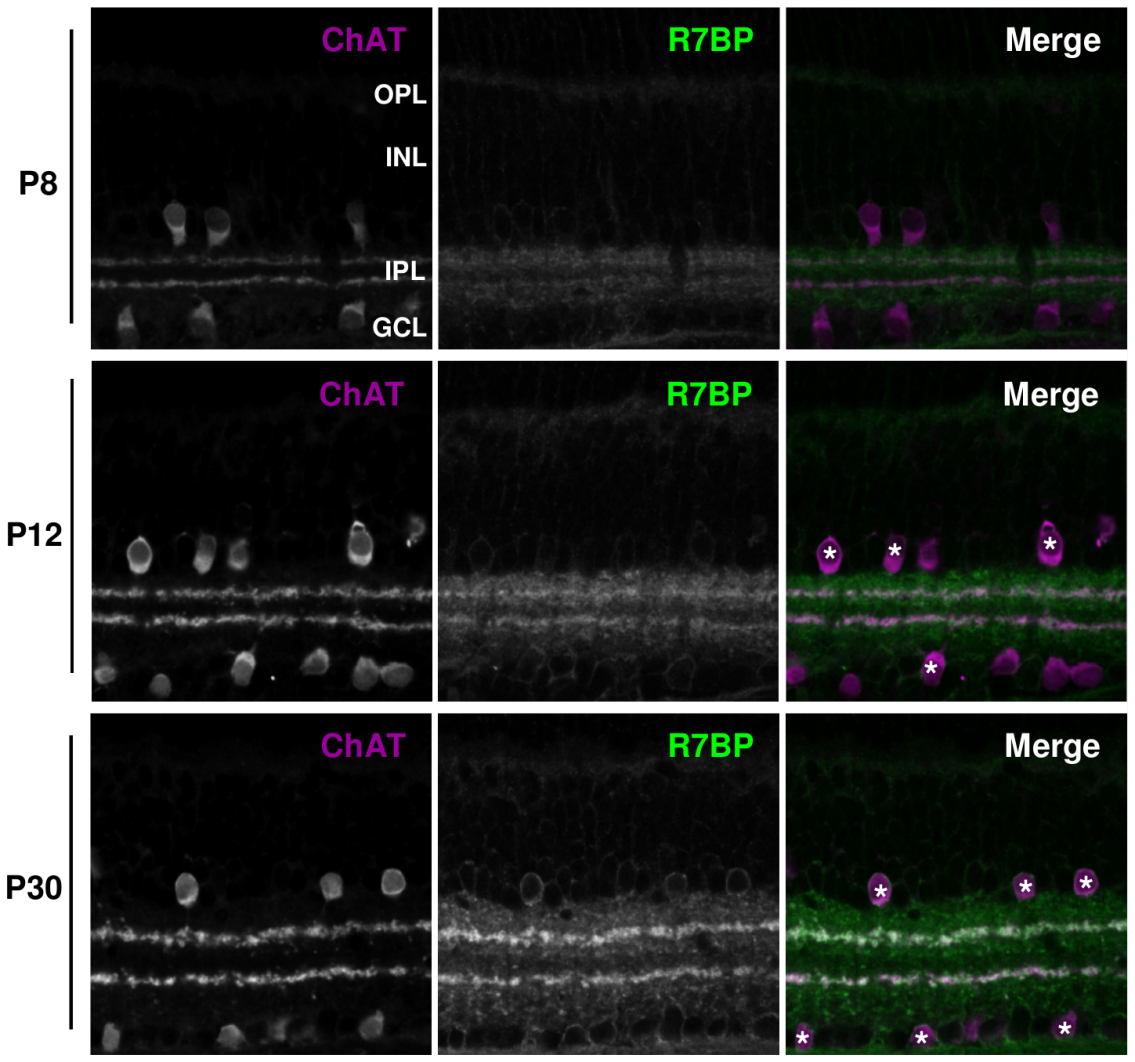
R7BP is induced during postnatal development. At P8, R7BP (green) is expressed in the OPL, INL, IPL, and GCL. SACs (ChAT-positive cells; magenta) are indicated. R7BP expression in retina is increased at P12 and P30 relative to P8. Asterisks indicate R7BP-positive, ChAT-positive SACs.

Next, we determined the expression pattern of R7BP during postnatal retinal development. This was examined because in brain the expression of R7BP/R7-RGS/Gβ5 complexes is induced postnatally during synaptic refinement [38], [47] and because R7-RGS/Gβ5 heterodimers are required for normal development and dendritic organization in cerebellum, hippocampus, and retina [23], [28], [48]. At P8, we detected specific R7BP staining in the IPL but not in the INL or GCL (Figure 2). At P12, R7BP staining in the IPL and S2/S4 was more intense, and became detectable in the OPL, INL and GCL. An adult pattern of R7BP expression was evident at P30. Thus, R7BP is expressed before photoreceptors mature and is refined as retinal development is completed.

### R7BP ablation does not disrupt outer retina function

**Figure 3:**
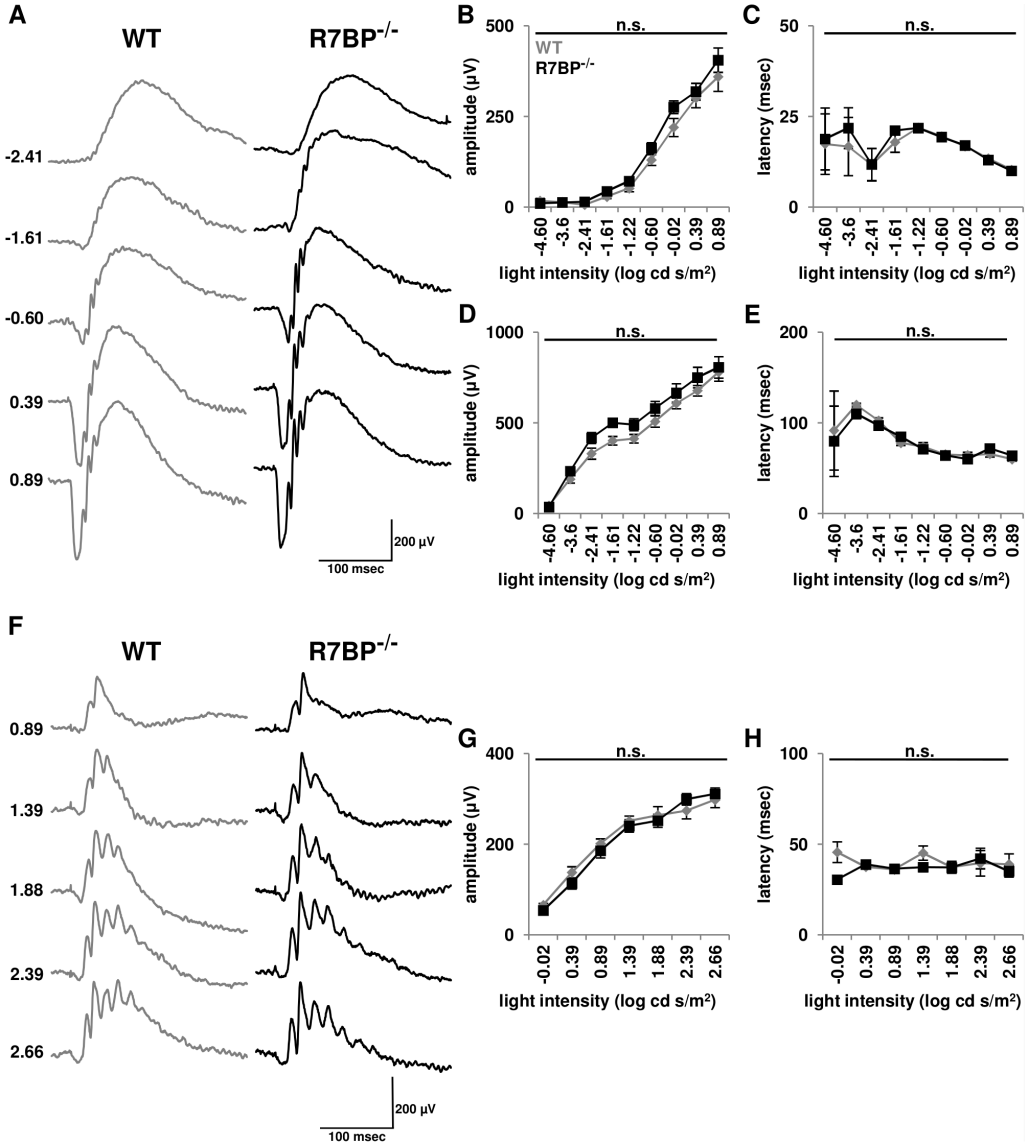
R7BP^−/−^ mice have essentially normal light-evoked photoreceptor and ON bipolar cell activity. **A.** Representative ERG responses from dark-adapted WT and R7BP^−/−^ mice (∼3 months) were obtained over the indicated range of flash intensities (log cd s/m^2^). **B-C.** Quantification of ERG a-wave peak amplitude (B) and latency (C) in adult dark-adapted WT (gray) and R7BP^−/−^ (black) mice. **D-E.** Ablation of R7BP does not alter dark-adapted b-wave peak amplitude (D) or latency (E). **F.** Representative ERG responses of light-adapted WT and R7BP^−/−^ mice over the photopic range of flash intensities (log cd s/m^2^). **G-H.** Similar ERG b-wave peak amplitude (G) and latency (H) in light-adapted WT and R7BP^−/−^ mice were observed. Error bars represent ±SEM.

Previous investigations have shown that R7BP^−/−^ mice exhibit normal retinal morphology and rod-driven light responses of ON bipolar cells [36]. To determine whether R7BP ablation affects rod- or cone-driven responses over a full range of stimulus intensities, we performed electroretinography of dark-adapted (Figure 3A) and light-adapted mice (Figure 3F). In dark-adapted WT and R7BP^−/−^ mice (n = 5), we observed no difference in a-wave amplitude or latency corresponding to light-evoked hyperpolarization of rods (low light intensities) or rods and cones (higher intensities) (Figure 3B/C). The ERG b-wave, which primarily reports depolarization of ON-bipolar cells, exhibited a trend in R7BP^−/−^ mice toward increased amplitude upon rod-specific stimulation but failed to reach the threshold for significance (Figure 3D). B-wave latency (time to b-wave peak after flash) was unaffected by the absence of R7BP (Figure 3E). Photopic (cone-specific) responses revealed by constant background illumination and high intensity flashes revealed no change in b-wave amplitude or latency (Figure 3G/H). Therefore, R7BP ablation had no significant effect on light-evoked responses in outer retina.

### R7BP ablation affects the firing rate and latency of ON RGCs driven by rods and cones but not by rods alone

**Figure 4:**
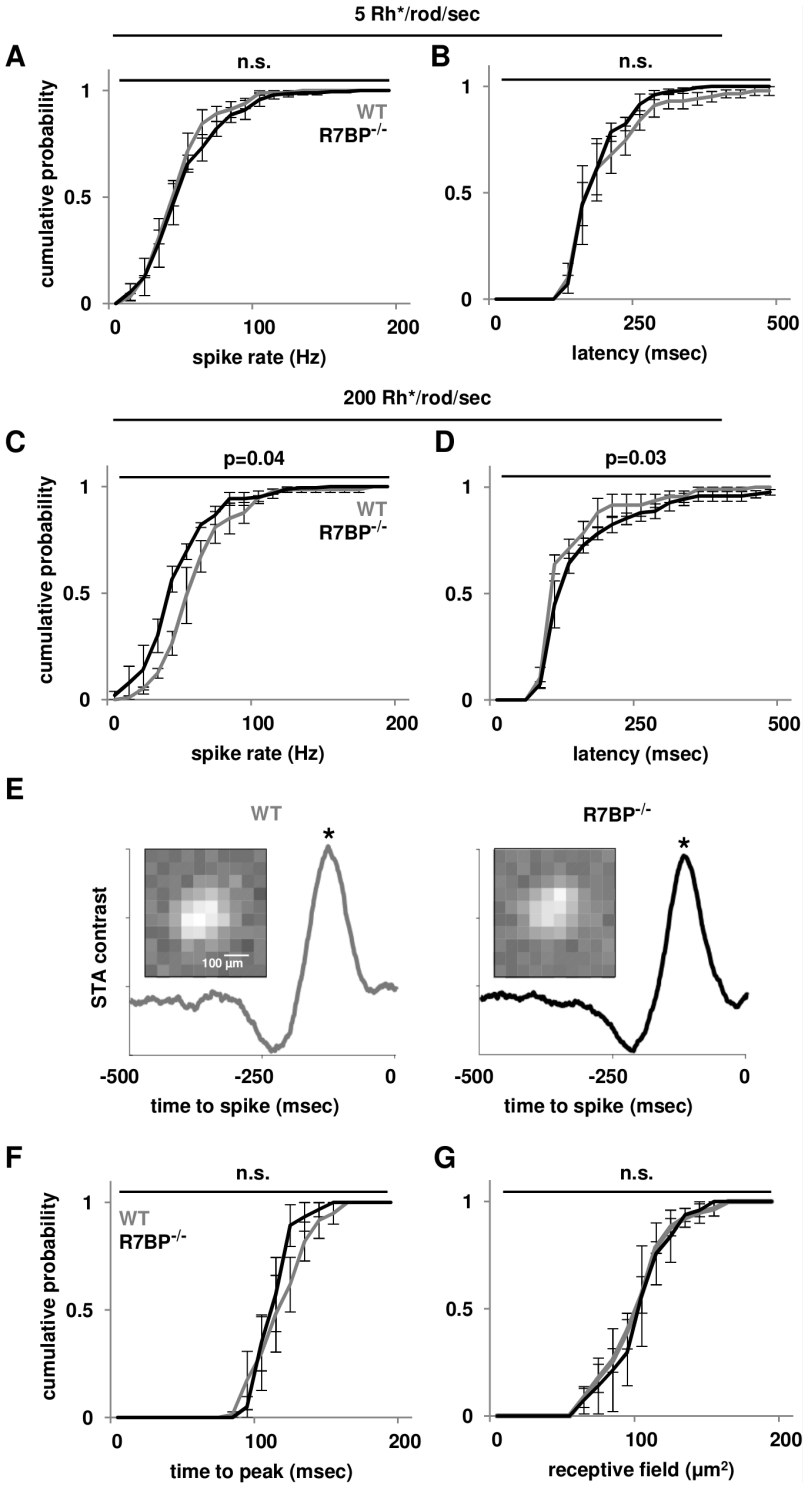
Ablation of R7BP alters mesopic but not scotopic ON RGC light responses. **A-B.** Distribution of mean spike rate (B) and latency (C) of P20-21 WT (gray lines) and R7BP^−/−^ (black lines) ON RGCs is similar under scotopic illumination. **C-D.** Ablation of R7BP decreases the mean spike rate (D) and increases the latency (E) of P20–21 ON RGCs under mesopic illumination. **E.** Representative spike trigger average (STA) stimuli of WT (gray) and R7BP^−/−^ (black) ON RGCs. Inset: Corresponding receptive field as revealed by images of STAs at peak contrast, asterisks. **F.** Time-to-peak between stimulus (STA peak contrast, asterisks) and response (spike) of WT and R7BP^−/−^ ON RGCs. **G.** Receptive field sizes of ON RGCs in P20-21 WT and R7BP^−/−^ retina were similar. Error bars represent ±SEM.

Having shown that outer retina function is essentially preserved in R7BP^−/−^ mice, we then analyzed inner retina function by performing multielectrode array (MEA) recordings of RGC activity. In dark-adapted retina (P20) we measured mean spike rates and latencies (time between flash and peak RGC firing rate) of transient ON RGCs under full field illumination at scotopic and mesopic intensities (5 and 200 Rh*/rod/sec, respectively). Under scotopic illumination, the absence of R7BP did not affect transient ON RGC mean spike rate (WT: 53±2 Hz, R7BP^−/−^: 54±2 Hz; mean±SEM, WT: n = 94, R7BP^−/−^: n = 150, Figure 4A) or latency (WT: 0.21±0.01 msec, R7BP^−/−^: 0.21±0.01 msec, Figure 4B). In contrast, mesopic illumination of rods and cones indicated that transient ON RGCs in R7BP^−/−^ retinas exhibited slower firing rates (WT: 59±3 Hz, R7BP^−/−^: 50±2 Hz, p<0.04; Figure 4C) and longer latency (WT: 0.14±0.01 msec, R7BP^−/−^: 0.19±0.02 msec, p<0.03, Figure 4D). R7BP ablation therefore affected light response of ON RGCs driven by mesopic but not scotopic illumination.

To characterize RGC light responses further we presented checkerboard Gaussian white noise illumination and calculated spike-triggered average (STA) stimuli through correlation of RGC spike trains to the patterns of light squares that evoked the spikes (see Methods for further description). Representative ON biphasic STAs are shown in Figure 4E (WT: n = 58, R7BP^−/−^: n = 63). The average size of WT and R7BP^−/−^ RGCs receptive fields were calculated as a two-dimensional Gaussian fit of the spatial profile (Figure 4E, insets) at the STA temporal maximum (peak contrast, asterisks). This analysis indicated that time to peak (difference between STA temporal maximum and spike; WT: 120±3 msec, R7BP^−/−^: 110±1 msec; mean±SEM; Figure 4F) and the average receptive field radius (WT: 110±3 µm; R7BP^−/−^: 110±2 µm; Figure 4G) of WT and R7BP^−/−^ ON RGCs were similar. Thus, whereas R7BP ablation did not affect the average size of ON RGC receptive fields, it did affect the light-evoked firing rate and latency of ON RGCs in response to full-field flashes.

### R7BP ablation alters the burst duration of glutamatergic waves in developing retina

Several considerations prompted us to investigate whether R7BP ablation affects spontaneous activity exhibited as propagating waves of correlated, high intensity firing of neighboring RGCs in developing retina (reviewed in [49]). In P0-P10 murine retina, cholinergic waves driven by acetylcholine release from SACs activate nicotinic acetylcholine receptors (nAChRs) on RGCs [50]. From P11 to ∼P16, cholinergic waves are replaced by glutamatergic waves in which ionotropic glutamate receptors on RGCs are stimulated by glutamate release from bipolar cells. Whether Gi/o-coupled receptor signaling regulated by R7-RGS proteins affects cholinergic or glutamatergic waves has not been investigated.

**Figure 5:**
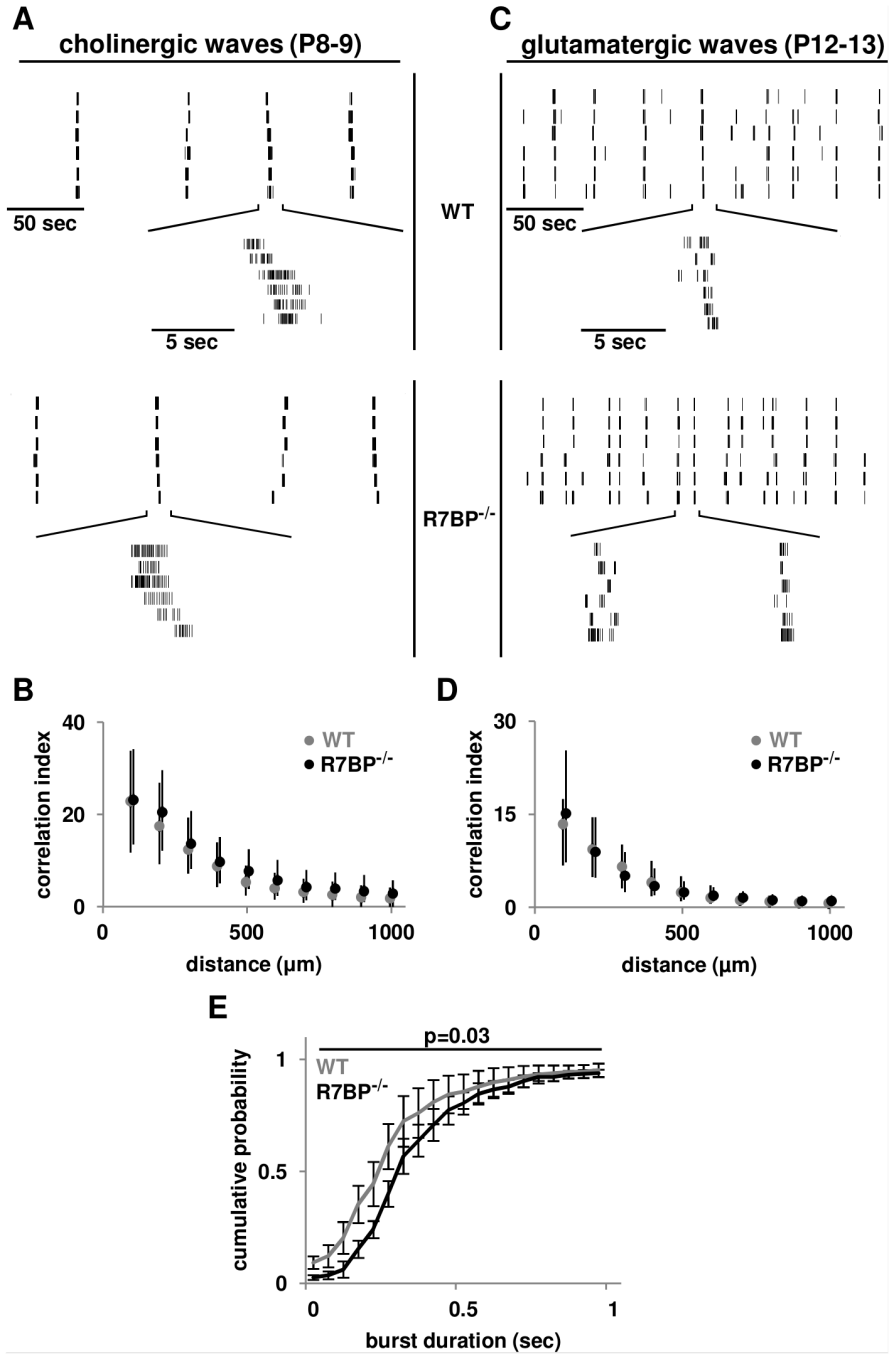
Ablation of R7BP increases glutamatergic wave burst duration. **A.** Representative raster plots of cholinergic waves from P8–9 WT and R7BP^−/−^ RGCs obtained by MEA recordings. **B.** Correlated firing of cholinergic waves is preserved in R7BP^−/−^ retina. Correlation indexes from P8-9 WT (gray) and R7BP^−/−^ (black) RGCs plotted as function of distance between the recording electrodes. Circles indicate the median. Lower and upper error bars indicate the 25th and 75th percentile, respectively. **C.** Representative raster plots of glutamatergic waves in P12–13 WT and R7BP^−/−^ RGCs. **D.** Correlated firing during glutamatergic waves is similar in WT (gray) and R7BP^−/−^ (black) retinas. **E.** Ablation of R7BP increases the burst duration of glutamatergic waves in P12-13 retina. Error bars represent ±SEM.

To address this question we used MEA recordings to analyze spontaneous waves in developing WT and R7BP^−/−^ retinas. Because R7BP is not expressed detectably in the retina until ∼P8, we analyzed spontaneous RGC activity in dark-adapted P8–9 and P12–13 retinas to examine cholinergic and glutamatergic waves, respectively. Several properties of spontaneous waves were measured, including firing rate, burst duration, interwave interval, and correlation indices. At P8–9, both WT and R7BP^−/−^ retinas exhibited correlated periodic bursting similar to previous descriptions of cholinergic waves [51]. Characteristic of glutamatergic waves, bursting at P12–13 in both WT and R7BP^−/−^ occurred more frequently and with shorter duration than at P8-9 (Figure 5A/C, Table 1). Thus, R7BP ablation apparently did not preclude transition between these two stages of waves. Furthermore, R7BP ablation did not affect cholinergic wave characteristics (Figure 5B, Table 1). Similarly, the correlation indices, spike rate, and interwave interval within glutamatergic waves were unaffected (Figure 5D, Table 1). However, burst duration of glutamatergic waves was longer in R7BP^−/−^ retina (WT: 0.41±0.01 sec, R7BP^−/−^: 0.55±0.02 sec, p<0.03, Figure 5E). Thus, R7BP-dependent regulation of R7-RGS proteins modulates distinct aspects of glutamatergic but not cholinergic waves in developing retina.

**Table 1:** Spatiotemporal properties of cholinergic and glutamatergic waves of WT and R7BP^−/−^ RGCs.

	P8–9		P12–13	
	WT	R7BP^−/−^	WT	R7BP^−/−^
**# of Neurons [Retinas]**	190 [3]	180 [3]	594 [4]	365 [4]
**Firing Rate (Hz)**				
Mean±SEM	0.21±0.01	0.22±0.01	0.44±0.02	0.37±0.02
25%	0.12	0.13	0.17	0.18
50%	0.19	0.20	0.32	0.30
75%	0.28	0.29	0.58	0.47
p Value	p = 0.75		p = 0.83	
**Burst Duration (sec)**				
Mean±SEM	0.94±0.04	0.95±0.04	0.41±0.01	0.55±0.02
25%	0.47	0.59	0.24	0.30
50%	0.80	0.89	0.30	0.42
75%	1.2	1.3	0.42	0.60
p Value	p = 0.79		p = 0.03*	
**Interwave Interval (sec)**				
Mean±SEM	60±3.5	68±3.1	22±8.1	23±8.1
25%	30	55	11	10
50%	54	75	20	21
75%	83	92	28	32
p Value	p = 0.67		p = 0.98	

Quantification of firing rate, burst duration, and interwave interval of cholinergic (P8–9) and glutamatergic (P12–13) retinal waves determined by MEA recordings.

### R7BP ablation does not impair segregation of retinogeniculate projections

**Figure 6:**
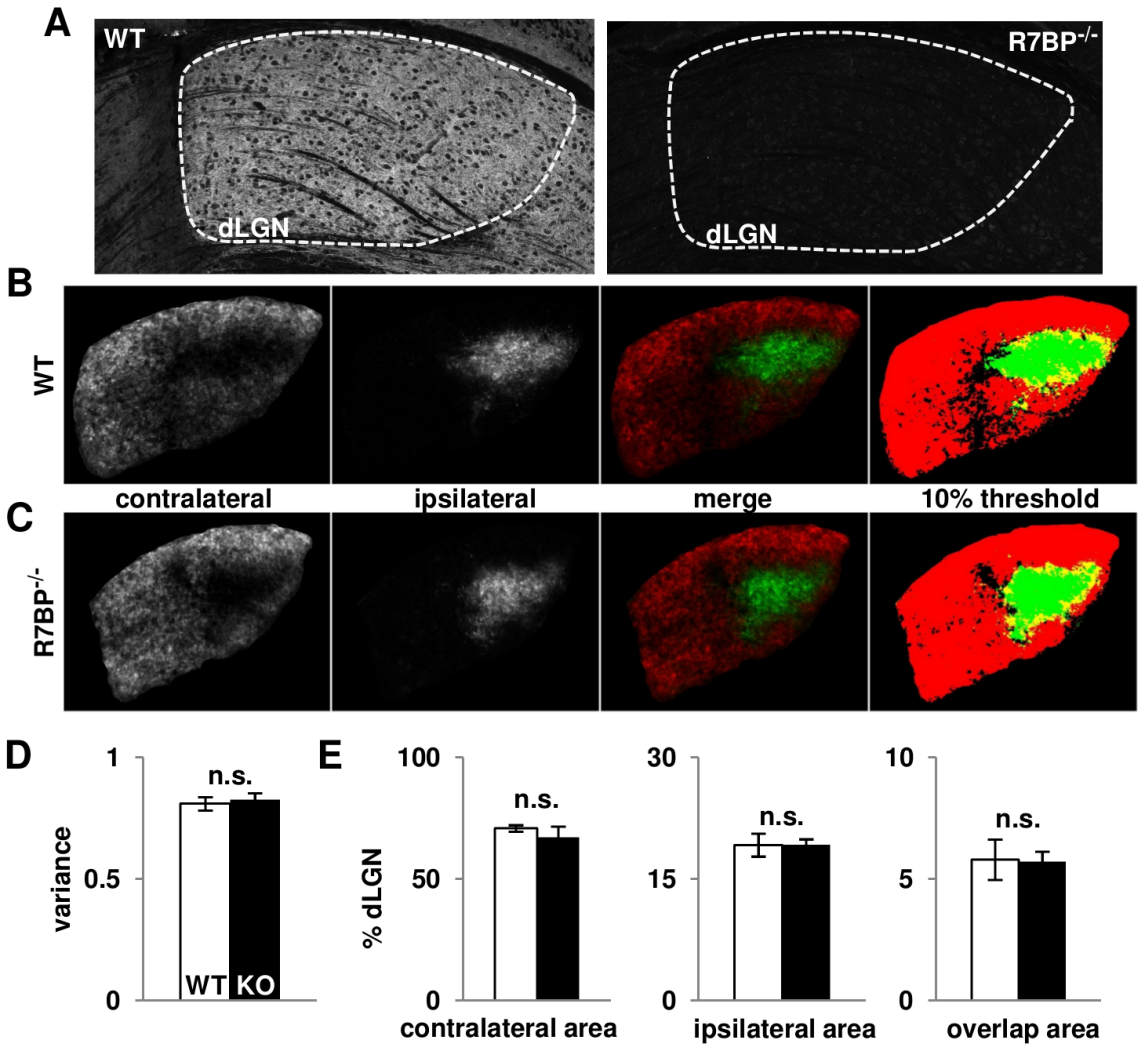
R7BP is expressed in the dLGN but is dispensable for segregation of retinogeniculate projections. **A.** R7BP is expressed in WT dorsal lateral geniculate nuclei (dLGN) (traced by dashed white line) but absent from R7BP^−/−^ dLGN. **B-C.** Representative fluorescent labeling of contralateral (red) and ipsilateral (green) retinogeniculate projections of P21 WT (B) or R7BP^−/−^ (C) dLGN using intraocularly injected, Alexa-conjugated cholera toxin B subunit (CTB) as anterograde tracers. Binarized images were thresholded at 10% to determine the area occupied by either contralateral (red), ipsilateral (green), or both (yellow) projections. **D.** Segregation of eye-specific domains is similar in WT (white bar) or R7BP^−/−^ (black bar) dLGN as measured by mean variance of R-value distributions from CTB-labeled retinogeniculate projections. **E.** Ablation of R7BP does not alter the area of dLGN innervated by contralateral, ipsilateral, or both projections. Error bars represent ±SEM.

Retinal waves play important roles in organizing axonal projections of RGCs that innervate eye-specific domains of the dorsal lateral geniculate nucleus (dLGN) of the thalamus (reviewed in [49], [52]). While it seemed unlikely that the modest change in glutamatergic wave burst duration observed in R7BP^−/−^ retina would be sufficient to impair retinogeniculate segregation, we found that R7BP is expressed highly in the adult dLGN (Figure 6A). This raised the possibility that loss of R7BP in the dLGN potentially could affect retinogeniculate organization by regulating activity of postsynaptic thalamocortical relay neurons.

To determine whether R7BP deficiency affects segregation of RGC projections into eye-specific domains in the dLGN, we injected Alexa 555 or Alexa 647 dye-conjugated cholera toxin B (CTB) as anterograde tracers into opposite eyes of WT and R7BP^−/−^ mice. Representative images of retinogeniculate labeling of the dLGN in WT and R7BP^−/−^ are shown in Figure 6B/C. Images quantified by analyzing variance of R-values (log_10_ ratio of ipsilateral and contralateral fluorescence signals on a pixel by pixel basis) revealed no detectable difference in retinogeniculate segregation in R7BP^−/−^ and WT littermates (Figure 6D). Similarly, areas occupied by contralateral, ipsilateral, or both projections were indistinguishable between WT and R7BP^−/−^ animals (Figure 6E). Thus, R7BP ablation in retina or dLGN was insufficient to disrupt retinogeniculate segregation.

### R7BP ^−/−^ mice exhibit normal spatial contrast sensitivity and visual acuity

To assess whether R7BP ablation affects overall visual perception, we evaluated optomotor responses as a means of determining visual acuity and contrast sensitivity under scotopic (-4.5 log cd/m^2^) and photopic (1.8 log cd/m^2^) conditions [43]. Results indicated that visual acuity in WT and R7BP^−/−^ mice (n = 5) was similar under both scotopic (WT: 0.34±0.03 cyc/deg, R7BP^−/−^: 0.35±0.03 cyc/deg; mean±SEM) and photopic illumination (WT: 0.54±0.02 cyc/deg, R7BP^−/−^: 0.50±0.04 cyc/deg). Similarly, no difference in contrast sensitivity was observed under scotopic (WT: 9.2±1.2, R7BP^−/−^: 11±2.8) or photopic illumination (WT: 48±6, R7BP^−/−^: 46±7). Thus, although R7BP deficiency affects light response of RGCs, it did not affect aspects of spatial vision required for normal optomotor reflexes.

## Discussion

Our analysis of R7BP^−/−^ mice indicates that Gi/o signaling evoked by endogenous transmitters and regulated by R7-RGS proteins modulates RGC activity in mature and developing inner retina. In mature retina, R7BP ablation had a modest but significant effect on mesopic transient ON RGC light responses, slowing the firing rate and increasing the latency, whereas ON RGC activity under scotopic illumination was unaffected. As R7BP is expressed most highly in inner retina and R7BP^−/−^ mice exhibit relatively normal outer retina structure [36] and function, these phenotypes apparently are consequences of inner retina dysfunction. Whether R7BP is functioning postsynaptically in RGCs or presynaptically in amacrine cells is unclear, as both cell types express R7BP. However, the effects of R7BP on light responses are consistent with evidence that RGC hyperpolarization can be regulated by activation of Gi/o-coupled receptors in either cell type. Indeed, activation of Gi/o-coupled A1 adenosine receptors can reduce RGC spiking by activating G-protein-coupled inwardly rectifying K^+^ (GIRK) and small conductance Ca^2+^-activated K^+^ (SK) channels [4]. Alternatively, activation of Gi/o-coupled group III mGluRs increases GABA release from amacrine cells and inhibitory drive experienced by RGCs [53].

In developing retina, ablation of R7BP increased the burst duration of RGCs driven by glutamatergic waves. This effect was specific for glutamatergic waves because loss of R7BP had no significant effect on cholinergic waves, consistent with low level expression of R7BP in the IPL and SACs at this stage of development (P8-9). The effect of R7BP ablation on glutamatergic wave burst duration in RGCs provides the first indication that regulation of Gi/o signaling by R7-RGS proteins modulates glutamatergic wave dynamics. Previous studies have identified corresponding roles for R7-RGS complexes in the developing nervous system, including ON BPC synapse formation [23], [28] and cerebral and hippocampal development [48].

Ultimately, these effects of R7BP ablation on ON RGC light response and glutamatergic waves were modest and insufficient to affect downstream processes including spatial vision and retinogeniculate segregation. Because R7BP deficiency apparently results in modest impairment of R7-RGS-mediated regulation of Gi/o signaling in inner retina, it may be necessary to eliminate R7-RGS proteins in inner retinal cell types to elicit pronounced phenotypes. This approach may reveal how augmented Gi/o signaling affects inner retina development and function. Studies of R7-RGS isoforms in SACs may be of particular interest because Gi/o-coupled receptors modulate SAC neurotransmitter release to control direction-selective (DS) circuits [54], [55].
